# A comparison of comorbidities obtained from hospital administrative data and medical charts in older patients with pneumonia

**DOI:** 10.1186/1472-6963-11-105

**Published:** 2011-05-18

**Authors:** Wai Fung Chong, Yew Yoong Ding, Bee Hoon Heng

**Affiliations:** 1 Health Services and Outcomes Research, National Healthcare Group, 6 Commonwealth Lane, #04-01/02 GMTI Building, Singapore 149547; 2 Department of Geriatric Medicine, Tan Tock Seng Hospital, 11 Jalan Tan Tock Seng Singapore 308433

## Abstract

**Background:**

The use of comorbidities in risk adjustment for health outcomes research is frequently necessary to explain some of the observed variations. Medical charts reviews to obtain information on comorbidities is laborious. Increasingly, electronic health care databases have provided an alternative for health services researchers to obtain comorbidity information. However, the rates obtained from databases may be either over- or under-reported. This study aims to (a) quantify the agreement between administrative data and medical charts review across a set of comorbidities; and (b) examine the factors associated with under- or over-reporting of comorbidities by administrative data.

**Methods:**

This is a retrospective cross-sectional study of patients aged 55 years and above, hospitalized for pneumonia at 3 acute care hospitals. Information on comorbidities were obtained from an electronic administrative database and compared with information from medical charts review. Logistic regression was performed to identify factors that were associated with under- or over-reporting of comorbidities by administrative data.

**Results:**

The prevalence of almost all comorbidities obtained from administrative data was lower than that obtained from medical charts review. Agreement between comorbidities obtained from medical charts and administrative data ranged from poor to very strong (kappa 0.01 to 0.78). Factors associated with over-reporting of comorbidities were increased length of hospital stay, disease severity, and death in hospital. In contrast, those associated with under-reporting were number of comorbidities, age, and hospital admission in the previous 90 days.

**Conclusions:**

The validity of using secondary diagnoses from administrative data as an alternative to medical charts for identification of comorbidities varies with the specific condition in question, and is influenced by factors such as age, number of comorbidities, hospital admission in the previous 90 days, severity of illness, length of hospitalization, and whether inhospital death occurred. These factors need to be taken into account when relying on administrative data for comorbidity information.

## Background

Pre-existing conditions or comorbidities have been used in risk adjustment for health outcomes research [1,2]. The number and type of comorbidities can have a significant impact on patient outcomes and may explain some of the observed variations [3-9]. Traditionally, medical charts were used to obtain information on comorbidities. This is a very laborious process. With the advent of electronic health care databases that capture financial data for the purpose of claims, such administrative data have provided an alternative for health services researchers to obtain comorbidity information for outcomes research [10-13].

While information obtained from administrative databases has been used for risk adjustment [14-16], the evidence on accuracy of secondary diagnoses from administrative databases as a substitute for comorbidities has been mixed [17-30]. Previous studies have found that administrative databases tend to under-report some comorbidities but overestimate the prevalence of others when compared with information from medical charts [17-21,24,28-31]. Comorbidities that showed better agreement between both data sources were solid tumor [17], diabetes mellitus [25], connective tissue disease [17,19], and chronic pulmonary disease [25]. The conditions with poor agreement were renal disease [17], dementia [17], hypertension [19], diabetes with complications [17,25] and peripheral vascular disease [20].

Reasons for the discordance of comorbidity assignment from both sources of information have been offered. Romano et al [24] and Powell et al [21] found that conditions which were asymptomatic tended to be under-reported in administrative data. Iezzoni et al [18] suggested that some acute medical conditions or complications were deemed by coders to be more important than others, thereby creating coding bias.

Humphries et al [25] found that although the agreement between comorbdities from two different sources as measured by kappa statistics was only fair, there was no significant difference in the predictive value for all-cause mortality in a group of patients who have undergone percutaneous coronary intervention. Newschaffer et al [29] found similar results in a population of patients with breast cancer. Van Doorn [32] showed the same findings in a population of older adults. Susser et al [33] found that the Charlson Comorbidity Index (CCI) [16] constructed with comorbidities obtained from either administrative data or self-report had similar predictive validity for functional decline and health services utilization.

To date, there has not been any study that identified patient characteristics associated with the likelihood of over- or under-reporting of comorbidities in administrative data, particularly in older populations where the prevalence of comorbidities is higher. This study aimed to (a) quantify the agreement between administrative data and medical charts review across a set of comorbidities in older hospitalized persons; and (b) examine the factors associated with under- or over-reporting of comorbidities by the administrative data. We hypothesized that patients with high number of comorbidities were more likely to have under-reporting of comorbidities in the administrative data, while those who had longer lengths of hospital stay were more likely to have over-reporting.

## Methods

### Study population

The study population comprised patients aged 55 years and above who were hospitalized for pneumonia between 1 January and 31 December 2007 at 3 acute care hospitals in Singapore. They were identified from hospital administrative data of the National Healthcare Group (NHG) Operations Data Store (ODS) through the coding classification of the Australian National Diagnosis Related Groups (AN-DRG) version 3.1. Those assigned to DRG170 (Respiratory infections/inflammations age > 54 with complications) and DRG171 (Respiratory infections/inflammations age > 54 without complications or age < 55 with complications) were included. In addition, patients with DRG003 (Tracheostomy except for mouth, larynx or pharynx disorder age >15) were checked against their respective International Classification of Diseases, 9^th ^Revision, Clinical Modification (ICD-9CM) codes for pneumonia. Those with primary ICD-9CM codes of 481, 482, 485, and 486 were also included. In Singapore, primary diagnosis refers to the reason for admission. Patients admitted for pneumonia were selected for this study as it is an acute medical condition, and not a comorbidity.

### Data collection

Information was obtained from two sources, namely medical charts and routine hospital administrative data. For medical charts, data was extracted from the emergency department notes, inpatient notes, and specialist consultation letters by a trained research nurse. Ten percent of the medical charts were reviewed by the author to check for consistency. A data collection form was used to record information on demographic characteristics (age, gender, and ethnic group), hospitalization (length of stay, comorbidities, and hospital admissions in the previous 90 days), physical examination (altered mental status, respiratory rate, and blood pressure) and selected laboratory data (serum urea level). For comorbidities, the set of 30 conditions listed by Elixhauser et al [34] was used, with the exception of Human Immunodeficiency Virus (HIV) infection because the medical charts of patients with HIV were not available for review. Only those documented by the attending physicians during the first 24 hours of admission were included to ensure that complications occurring during the course of the hospital stay were excluded. The research nurse who performed data extraction from medical charts was blinded to information from the hospital administrative data.

For administrative data, only secondary codes for each index admissions selected were used without 'looking back' at previous admissions. Secondary or additional ICD-9CM codes were extracted from the administrative databases and mapped to the 29 comorbidities. These ICD-9CM codes were entered into the hospital administrative databases by trained clinical coders after patients were discharged. There was no limit to the number of codes in the secondary diagnoses field. The clinical coders in the hospitals were a mix of non-practicing physicians as well as professionally trained coders with clinical background (nursing or allied health). All clinical coders were trained by expert coders and the coding practice adheres to the Singapore Coding Directive, a national coding standard. The coding accuracy is monitored through periodic and stringent audits by the Ministry of Health of Singapore for the purpose of funding/reimbursement that is DRG-based and hence dependent on comorbities reported. All comorbidities reflected by secondary ICD-9CM codes in the routine administrative data were included.

A total of 29 comorbidities were included. Comorbidities derived from medical charts were considered the reference ("gold standard"). Each comorbidity derived from administrative database was re-coded to specify if it was under-reported or over-reported compared to the "gold standard". The total number of comorbidity comparisons was the number of hospital episodes multiplied by the 29 comorbidities.

Mortality is an important outcome and an indicator of quality of care for patients with pneumonia. Prediction tools are used to predict and stratify patients' mortality risk and a means for deciding on the course of clinical management. For this study, selected clinical data to construct the CURB (**C**onfusion, **U**rea > 7 mmol/L, **R**espiratory rate > 30/min, **B**lood pressure with low systolic <90 or diastolic <60 mmHg) score to stratify pneumonia severity by risk of death [35] were also collected. A score of 0-1 predicts lowest risk of mortality and a score of 4 predicts the highest risk.

### Data analysis

The unit of analysis was hospital episode. Agreement between the two sources of data was quantified using Cohen's kappa coefficient, κ. Kappa value above 0.75 indicates an excellent level of agreement beyond chance, 0.40 through 0.75 represent fair to good agreement, and kappa value less than 0.4 indicates poor agreement [36]. Sensitivity, specificity, positive predictive value (PPV) and negative predictive value (NPV) were calculated for each comorbid condition. Sensitivity was defined as the proportion of medical charts with documentation of the comorbidity that was also coded with that comorbidity in the hospital administrative data. Hospital episodes where comorbidities that were documented in the medical charts but were not coded in the hospital administrative data were assigned as under-reporting. Specificity was defined as the proportion of medical charts without documentation of the comorbidity which was not coded with that condition in the hospital administrative data too. Hospital episodes where comorbidities that were not documented in the medical charts but were coded in the hospital administrative data were assigned as over-reporting.

Multinomial regression was performed to identify factors that were associated with over-reporting of comorbidities for all comparisons, followed by for each of the 29 comorbidities in turn. This process was repeated for under-reporting of comorbidities. The independent variables included age, gender, disease severity, number of comorbidities, previous hospital admissions within the last 90days, length of hospital stay, and inhospital death. Odds ratios for these factors were obtained for both over- and under-reporting with the reference being same-coding. Hierarchical multinomial regression modeling was performed for these analyses using the STATA program for generalized linear latent and mixed models (GLLAMM) [37]. This is to account for the effect of clustering due to multiple hospital episodes for the same patient during the study period.

All statistical tests were carried out using the STATA version 9.2 (Stata Corp, College Station, Texas). Statistical significance was taken at p-values of less than 0.05.

The study was approved by the National Healthcare Group's Institutional Review Board.

## Results

**Figure 1:**
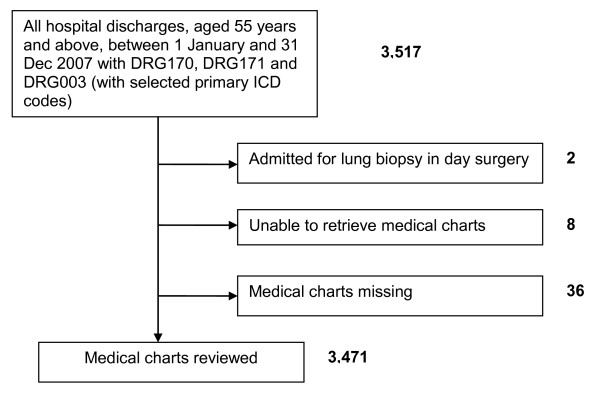
**Study flow diagram**.

A total of 3517 hospital admissions for pneumonia that satisfied the criteria for the study population were identified. Of these, 46 admissions (1.3%) were excluded from the study as their medical charts were not available during the period of review (Figure 1). The characteristics of the remaining 3471 admissions are summarized in Table 1.

**Table 1:** Patient characteristics (n = 3471)

Characteristic	
Mean age, years (SD)	77.3 (10.8)

Male gender, n (%)	1885 (54.3)

Nursing home residents, n (%)	675 (19.5)

Ethnic group, n (%)	
Chinese	2721 (78.4)
Malay	373 (10.7)
Indian	225 (6.5)
Others	152 (4.4)

No. of Elixhauser comorbid conditions, n (%)	
0	258 (7.4)
1-2	1544 (44.5)
3-4	1263 (36.4)
5-6	367 (10.6)
7-8	34 (1.0)
9-10	5 (0.1)
>10	0 (0)

Hospital admission in the previous 90 days, n (%)	440 (12.7)

CURB* score, n (%)	
0	1544 (44.5)
1	1399 (40.3)
≥2	528 (15.2)

Mean length of stay, days (SD)	9.8 (12.4)

Death in hospital, n (%)	693 (20.0)

*CURB = Constructed from 4 "core" adverse prognostic features: **C**onfusion, **U**rea, **R**espiratory rate, **B**lood pressure [35]

Table 2 shows the prevalence of the comorbidities obtained from medical charts and administrative data, and the agreement between both data sources. The prevalence of comorbidities obtained from medical charts review and administrative data ranged from 0.1 to 59.6 percent and 0.3 to 34.3 percent, respectively. The prevalence rates of all comorbidities derived from medical charts were higher than that obtained from routine administrative databases except for diabetes with complications, coagulopathy, deficiency anemias, weight loss, blood loss anemia, and paralysis. Drug abuse and obesity were not coded in the administrative data for any of the cases reviewed. The number of secondary diagnoses coded in the administrative database for each case range from 0 to 21, with a median of 6 diagnoses.

**Table 2:** Prevalence of comorbidities and agreement between data derived from medical charts and administrative data (n = 3471)

Comorbidities	Medical charts No. (%)	Administrative data No. (%)	Kappa (95% CI)
Diabetes, uncomplicated	996 (28.7)	900 (25.9)	0.78 (0.77 - 0.80)

Metastatic cancer	116 (3.3)	112 (3.2)	0.71 (0.70 - 0.73)

Diabetes, complicated	203 (5.8)	256 (7.4)	0.69 (0.67 - 0.71)

Chronic pulmonary disease	561 (16.2)	417 (12.0)	0.54 (0.53 - 0.56)

Lymphoma	20 (0.6)	17 (0.5)	0.54 (0.52 - 0.56)

Alcohol abuse	33 (1.0)	23 (0.7)	0.53 (0.52 - 0.55)

Hypertension	2068 (59.6)	1190 (34.3)	0.45 (0.44 - 0.47)

Peripheral vascular disorders	199 (5.7)	94 (2.7)	0.44 (0.43 - 0.46)

Liver disease	109 (3.1)	57 (1.6)	0.43 (0.42 - 0.45)

Congestive heart failure	469 (13.5)	252 (7.3)	0.40 (0.39 - 0.42)

RA/Collagen vascular disease	43 (1.2)	12 (0.3)	0.36 (0.35 - 0.37)

Cardiac arrhythmias	489 (14.1)	222 (6.4)	0.34 (0.33 - 0.36)

Coagulopathy	118 (3.4)	271 (7.8)	0.33 (0.32 - 0.35)

Hypothyroidism	118 (3.4)	40 (1.2)	0.33 (0.32 - 0.35)

Other neurological disorders	426 (12.3)	142 (4.1)	0.33 (0.31 - 0.34)

Solid tumor without metastasis	363 (10.5)	200 (5.8)	0.32 (0.30 - 0.34)

Fluid and electrolyte disorders	942 (27.1)	712 (20.5)	0.32 (0.30 - 0.33)

Deficiency anemias	474 (13.7)	704 (20.3)	0.28 (0.26 - 0.29)

Valvular disease	98 (2.8)	38 (1.1)	0.21 (0.19 - 0.22)

Pulmonary circulation disorders	44 (1.3)	16 (0.5)	0.20 (0.18 - 0.21)

Psychoses	97 (2.8)	13 (0.4)	0.14 (0.13 - 0.15)

Weight loss	18 (0.5)	82 (2.4)	0.13 (0.12 - 0.15)

Depression	319 (9.2)	33 (1.0)	0.08 (0.07 - 0.09)

Renal failure	447 (12.9)	24 (0.7)	0.06 (0.05 - 0.07)

Peptic ulcer disease	174 (5.0)	16 (0.5)	0.05 (0.04 - 0.05)

Blood loss anemia	3 (0.1)	57 (1.6)	0.03 (0.02 - 0.04)

Paralysis	16 (0.5)	128 (3.7)	0.01 (-0.01 - 0.02)

The overall agreement between both data sources using kappa statistics ranged widely from 0.01 (poor agreement) to 0.78 (excellent agreement). Diabetes mellitus (uncomplicated and complicated), metastatic cancer, chronic pulmonary disease, lymphoma and alcohol abuse reported the highest kappa statistic values of 0.53 to 0.78.

Table 3 shows the sensitivity, specificity, positive predictive value and negative predictive values. There were 6 comorbidities with sensitivity of more than 50 percent. Those that were most under-reported were peptic ulcer disease, renal failure, depression, paralysis and psychoses, with sensitivity of less than 9 percent. The specificity of the comorbidities was good, with only 6 comorbidities having specificity of less than 96%. These conditions were deficiency anemias, fluid and electrolyte disorders, coagulopathy, hypertension, chronic pulmonary disease, and uncomplicated diabetes, and represent conditions most over-reported in the administrative data. The positive predictive values (PPV) for the comorbidities ranged from 0.8 to 94.4 percent. The negative predictive values (NPV) for all the comorbidities were more than 80 percent, except for hypertension (58.6%).

**Table 3:** Sensitivity, specificity, positive predictive value (PPV) and negative predictive value (NPV) of comorbidities (n = 3471)

Comorbidities	Sensitivity		Specificity		Positive Predictive Value		Negative Predictive Value	
	
	%	95% CI	%	95% CI	%	95% CI	%	95% CI
Diabetes, complicated	80.3	74.2 - 85.5	97.2	96.5 - 97.7	63.7	57.5 - 69.6	98.8	98.3 - 99.1

Diabetes, uncomplicated	80.1	77.5 - 82.6	95.9	95.0 - 96.6	88.7	86.4 - 90.7	92.3	91.2 - 93.3

Metastatic cancer	70.7	61.5 - 78.8	99.1	98.7 - 99.4	73.2	64.0 - 78.8	99.0	98.6 - 99.3

Coagulopathy	60.2	50.8 - 69.1	94.0	93.2 - 94.8	26.2	21.1 - 31.9	98.5	98.1 - 98.9

Hypertension	54.3	52.1 - 56.5	95.2	94.0 - 96.3	94.4	92.9 - 95.6	58.6	56.5 - 60.6

Chronic pulmonary disease	52.8	48.5 - 57.0	95.8	95.1 - 96.5	71.0	66.4 - 75.3	91.3	90.3 - 92.3

Lymphoma	50.0	27.2 - 72.8	99.8	99.6 - 99.9	58.8	32.9 - 81.6	99.7	99.5 - 99.9

Deficiency anemias	49.2	44.6 - 53.8	84.3	82.9 - 85.6	33.1	29.6 - 36.7	91.3	90.2 - 92.3

Alcohol abuse	45.5	28.1 - 63.7	99.8	99.5 - 99.9	65.2	42.7 - 83.6	99.5	99.2 - 99.7

Fluid and electrolyte disorders	41.8	38.7 - 45.1	87.4	86.1 - 88.7	55.3	51.6 - 59.0	80.1	78.6 - 81.6

Weight loss	38.9	17.3 - 64.3	97.8	97.3 - 98.3	8.5	3.5 - 16.8	99.7	99.4 - 99.8

Congestive heart failure	35.4	31.1 - 39.9	97.1	96.5 - 97.7	65.9	59.7 - 71.7	90.6	89.5 - 91.6

Peripheral vascular disorders	34.2	27.6 - 41.2	99.2	98.8 - 99.5	72.3	62.2 - 81.1	96.1	95.4 - 96.8

Liver disease	33.9	25.2 - 43.6	99.4	99.1 - 99.6	64.9	51.1 - 77.1	97.9	97.4 - 98.4

Blood loss anemia	33.3	0.8 - 90.6	98.4	97.9 - 98.8	1.8	0.0 - 9.4	99.9	99.8 - >99.9

Cardiac arrhythmias	29.0	25.1 - 33.3	97.3	96.7 - 97.9	64.0	57.3 - 70.3	89.3	88.2 - 90.4

Solid tumor without metastasis	28.7	24.1 - 33.6	96.9	96.2 - 97.5	52.0	44.8 - 59.1	92.1	91.1 - 93.0

Other neurological disorders	24.7	20.6 - 29.0	98.8	98.3 - 99.1	73.9	65.9 - 80.9	90.4	89.3 - 91.3

Rheumatoid Arthritis/Collagen vascular disease	23.3	11.8 - 38.6	99.9	99.8 - >99.9	83.3	51.6 - 97.9	99.1	98.7 - 99.3

Hypothyroidism	22.9	15.7 - 31.5	99.6	99.3 - 99.8	67.5	50.9 - 81.4	97.4	96.8 - 97.9

Valvular disease	15.3	8.8 - 24.0	99.3	99.0 - 99.6	39.5	24.0 - 56.6	97.6	97.0 - 98.1

Pulmonary circulation disorders	13.6	5.2 - 27.4	99.7	99.5 - 99.9	37.5	15.2 - 64.6	98.9	98.5 - 99.2

Psychoses	8.3	3.6 - 15.6	99.9	99.7 - >99.9	61.5	31.6 - 86.1	97.4	96.8 - 97.9

Paralysis	6.3	0.2 - 30.2	96.3	95.6 - 96.9	0.8	0.0 - 4.3	99.6	99.3 - 99.8

Depression	5.0	2.9 - 8.0	99.5	99.1 - 99.7	48.5	30.8 - 66.5	91.2	90.2 - 92.1

Renal failure	3.8	2.2 - 6.0	99.8	99.5 - 99.9	70.8	48.9 - 87.4	87.5	86.4 - 88.6

Peptic ulcer disease	2.9	0.9 - 6.6	99.7	99.4 - 99.8	31.3	11.0 - 58.7	95.1	94.3 - 95.8

There were 100,659 comorbidity comparisons available for analyses. In the multinomial regression analysis, factors that were significantly associated with over-reporting were increased length of stay, disease severity and inhospital death. Factors that were significant for under-reporting were number of comorbidities, age and hospital admission in the previous 90 days (Table 4).

**Table 4:** Multinomial regression to determine factors that are associated with over-reporting and under-reporting of comorbidity (n = 100659)

Explanatory variable	Odds Ratio (95% confidence interval)	
	
	Over-reporting vs. same-reporting	Under-reporting vs. same-reporting
Age (per year)	1.003 (0.999 - 1.007)	1.010 (1.007 - 1.013)*

Male gender	1.009 (0.924 - 1.103)	1.057 (0.997 - 1.121)

Hospital admission in the previous 90 days	0.974 (0.879 - 1.079)	1.108 (1.039 - 1.182)*

Number of co-morbid conditions	1.013 (0.984 - 1.044)	1.443 (1.418 - 1.469)*

CURB score:		
0 (reference)	1.000	1.000
1	1.352 (1.219 - 1.500)*	1.109 (1.035 - 1.187)*
≥ 2	1.488 (1.303 - 1.700)*	1.058 (0.966 - 1.158)

Length of hospital stay (per day)	1.013 (1.011 - 1.015)*	1.000 (0.997 - 1.002)

Death in hospital	1.028 (1.118 - 1.379)*	1.028 (0.958 - 1.103)

* Statistical significance at p-value < 0.05

Increasing number of concomitant comorbidities and inhospital death increased the likelihood of over-reporting for several out of 10 individual comorbidities among the Elixhauser list (Table 5). Higher number of concurrent comorbidities consistently increased the likelihood of under-reporting across all the selected comorbidities, while increasing age and hospitalization in the previous 90 days did so for several of them (Table 6).

**Table 5:** Factors associated with over-reporting of 10 selected comorbidities

	Age	Previous admission	No of comorbidities	Length of stay	Severity of illness	Death
Metastatic cancer	+	+				+

Solid tumour without mestastases		+				+

Chronic heart failure	+		+	+		+

Renal failure			+			+

Chronic pulmonary disease						+

Peripheral vascular disease			+			+

Hypertension	+					

DM uncomplicated			+		+	

DM complicated			+			

Deficiency anaemia	+			+	+	

+: denotes a positive association that is statistically significant (p-value < 0.05)

**Table 6:** Factors associated with under-reporting of 10 selected comorbidities

	Age	Previous admission	Male	No of comorbidities	Length of stay	Severity of illness
Solid tumour without mestastases		+		+		

Liver disease	+	+		+		

Chronic heart failure	+		+	+	+	

Renal failure		+	+	+	+	+

Chronic pulmonary disease	+			+		

Peripheral vascular disease		+		+		

Hypertension	+			+		

DM uncomplicated				+	+	+

DM complicated				+		

Deficiency anaemia	+			+		

+: denotes a positive association that is statistically significant (p-value < 0.05)

Hierarchical modeling obtained very similar odds ratios and their 95% confidence intervals for factors explaining over- and under-reporting of comorbidities by the administrative data.

## Discussion

To the best of our knowledge, this is the first study that has evaluated the reliability of secondary diagnoses in the administrative data as a surrogate for comorbidities in Singapore. The results were consistent with other studies where the prevalence of comorbidities obtained from administrative data was lower than that obtained from medical charts for most conditions [17,19-21,24,38-40]. This is despite the fact that both the research nurse who abstracted information from medical charts and the clinical coders responsible for assigning the ICD-9CM codes in the administrative database had obtained their information from the same source. The discordance can be explained by examining the reasons for which the information was collected. Pre-existing conditions were documented in medical charts to assist clinicians in clinical care decisions. Secondary diagnoses codes from administrative data do not differentiate between pre-existing conditions or complications that occurred during the hospitalization [41]. There is no flag in the administrative data indicating that the conditions existed on or after admission. Therefore, conditions such as fluid and electrolyte disorders, deficiency anemias, blood loss anemia, diabetes with complications, and coagulopathy reported higher prevalence rates in administrative data than in medical charts because it is highly likely that these conditions arose during hospitalization as a result of worsening medical condition, complications of treatment, or confirmation of new medical conditions through laboratory tests. Clinical coders extract information for reimbursement purposes, and were more likely to include any conditions that have an impact on the utilization of resources during the episode of hospitalization. Hence, these conditions would be coded as secondary diagnoses in the administrative data, whilst the research nurse abstracting the information from the medical charts would have excluded them. If these conditions were used in risk adjustment to compare outcomes, these could potentially cause an "over-adjustment" of risk [42-48]. On the other hand, chronic conditions such as depression, psychoses, peptic ulcer disease, paralysis, or renal failure [49] were under-reported. These conditions may not have been active problems during hospitalization for pneumonia and therefore, did not contribute to increased resource utilization. It is most likely that the patients with history of drug abuse as documented in the medical charts, were no longer receiving treatment for it, and the condition would not be coded by the clinical coders. Similarly, obesity was not coded as a secondary diagnosis as the patients were not likely to have received treatment for obesity during their hospitalization for pneumonia.

The kappa statistics for diabetes (uncomplicated and complicated), metastatic cancer and COPD showed substantial agreement, as was found in other studies [17,21,25]. Similarly, Quan and colleagues [40] reported that comorbidities obtained from ICD-9-CM data had sensitivity ranging from 9.3% to 83.1%. The wide range for PPV was a reflection of the different prevalence rates for individual comorbidities. Paralysis, blood loss anemia and weight loss with the lowest PPV had the lowest prevalence in the medical charts as well.

Factors associated with over-reporting were length of hospitalization, severity of illness, and inhospital death. This is not unexpected as these factors were likely to be associated with an increase in the number of investigations and interventions during the hospital episode, resulting in identification of additional concurrent conditions and complications. These conditions were more likely to be coded as secondary diagnoses in the administrative data because they were related to increased resource utilization. Other researchers have previously found that some medical conditions or complications of treatment were judged more important than other chronic conditions when patients were critically ill or when they died [18,21,23,50].

Age, previous hospital admission and number of comorbidities were associated with under-reporting of comorbidities in the administrative database., Although there is no limit to the number of secondary diagnoses that can be coded, having a higher number of concurrent comorbidities may result in less important comorbidities being disregarded. Advancing age [8] and recent hospitalization are themselves associated with increased number of comorbidities. The association of these two factors with under-reporting may be a reflection of residual confounding of number of comorbidities that is not accounted for due to the specification of our regression models.

For the individual comorbidities, the same factors associated with over- or under-reporting for the whole study population were represented for most of the important ones we selected for detailed analyses. This was most clearly seen with number of concurrent comorbidities being associated with under-reporting for all 10 conditions. For length of hospitalization, severity of illness, inhospital death, age, and hospital admission in the previous 90 days, smaller sample sizes or lack of true effect may have accounted for the absence of association observed for some comorbidities. Nevertheless, we argue that the overall picture supports the results for the whole study population.

The main strength of this study is its large sample size. In addition, as there is no limit to the number of secondary diagnoses that can be coded in the administrative data, it is unlikely that the lower prevalence of comorbidities in the administrative data were due to restrictions imposed by the prevailing health information system.

There are several limitations of the study. Firstly, the findings of this study may not be generalized to older patients hospitalized for other acute illnesses because we only studied those with pneumonia. The use of DRG codes for pneumonia to identify cases could have included cases that did not meet the BTS criteria for pneumonia as DRG codes were assigned to reflect resource utilization. However, as the main objectives of this study were to compare the comorbidities obtained from two sources, and to identify the possible factors that may be associated with under or over-reporting of the comorbidities, and not on the outcomes for patients with pneumonia, the inclusion of such was not likely to have affected the findings. Secondly, documentation of comorbidities in the medical charts within the first 24 hours of admission may be incomplete in a busy ward environment with many patients waiting to be reviewed and therefore, may not be the ideal "gold standard" where comorbidities are concerned. To address this, we ensured that the research nurse was familiar with documentation in the medical charts and was trained to abstract very specific conditions. Attending physicians also had access to an electronic medical records system that contained information on known comorbidities. Therefore, we believe that the likelihood of documentation of important comorbidities being omitted is low. Thirdly, this study involved documentation of comorbidities in a single health care system and may not necessarily mirror the situation in other systems. Further research on factors associated with under- and over-reporting of comorbidities by administrative data in other health systems is needed to confirm our findings. Fourthly, we acknowledge that there may be other factors that could be associated with over- or under-reporting of the comorbidities but were not measured in our study. Although there were little prior research on this subject to guide us, we have included plausible factors on the basis of clinical opinion.

## Conclusions

Our study confirmed that the prevalence of almost all comorbidities obtained from administrative data was lower than that obtained from medical chart review. The validity of secondary diagnoses from administrative data varies with the specific comorbidity in question, and is influenced by factors such as age, number of comorbidities, hospital admission in the preceding 90 days, severity of illness, length of hospitalization, and whether inhospital death occurred. While some comorbidities were reported as secondary diagnoses with a reasonable level of accuracy, there were several that may not be used interchangeably. Researchers should be cautious and take these findings into account when using this source of information as an alternative to medical chart reviews for the purpose of measuring comorbidity burden. This may also affect policy decisions by hospital administrators as it may underestimate the true burden of illnesses. Further research is needed to confirm our findings in other patient populations.

## Competing interests

The authors declare that they have no competing interests.

## Authors' contributions

WFC carried out the study and drafted the manuscript. YYD conceived the study, participated in the study design and performed the statistical analysis. BHH participated in the design of the study and helped to draft the manuscript. All authors read and approved the final manuscript.

## Pre-publication history

The pre-publication history for this paper can be accessed here:

http://www.biomedcentral.com/1472-6963/11/105/prepub
